# Biochemical and biophysical characterization of the main protease, 3-chymotrypsin-like protease (3CLpro) from the novel coronavirus SARS-CoV 2

**DOI:** 10.1038/s41598-020-79357-0

**Published:** 2020-12-17

**Authors:** Juliana C. Ferreira, Wael M. Rabeh

**Affiliations:** Science Division, New York University Abu Dhabi, PO Box 129188, Abu Dhabi, United Arab Emirates

**Keywords:** Biochemistry, Biophysical chemistry

## Abstract

Severe acute respiratory syndrome-coronavirus 2 (SARS-CoV-2) is responsible for the novel coronavirus disease 2019 (COVID-19). An appealing antiviral drug target is the coronavirus 3C-like protease (3CLpro) that is responsible for the processing of the viral polyproteins and liberation of functional proteins essential for the maturation and infectivity of the virus. In this study, multiple thermal analytical techniques have been implemented to acquire the thermodynamic parameters of 3CLpro at different buffer conditions. 3CLpro exhibited relatively high thermodynamic stabilities over a wide pH range; however, the protease was found to be less stable in the presence of salts. Divalent metal cations reduced the thermodynamic stability of 3CLpro more than monovalent cations; however, altering the ionic strength of the buffer solution did not alter the stability of 3CLpro. Furthermore, the most stable thermal kinetic stability of 3CLpro was recorded at pH 7.5, with the highest enthalpy of activation calculated from the slope of Eyring plot. The biochemical and biophysical properties of 3CLpro explored here may improve the solubility and stability of 3CLpro for optimum conditions for the setup of an enzymatic assay for the screening of inhibitors to be used as lead candidates in the discovery of drugs and design of antiviral therapeutics against COVID-19.

## Introduction

The novel coronavirus disease 2019 (COVID-19) is an upper and lower acute respiratory tract infection that is caused by severe acute respiratory syndrome-coronavirus 2 (SARS-CoV-2), with symptoms ranging from mild to lethal^1–4^. SARS-CoV-2 is a member of the beta-coronavirus genus of the Coronavirinae subfamily that consists of four genuses (Alpha-, Beta-, Gamma-, and Delta-coronavirus)^5–8^. The Betacoronavirus genus contains four lineages A, B, C, and D, with lineage B containing SARS-CoV-2 as well as SARS-CoV that is responsible for the 2003 severe acute respiratory syndrome (SARS) with a case fatality rate of 14–15%, and the Middle East respiratory syndrome coronavirus (MERS-CoV) with a high case fatality rate of ~ 34.3% (statistic obtained from WHO, the World Health Organization) that is part of lineage C Betacoronavirus.

SARS-CoV-2 is part of Coronavirinae subfamily with one of the largest positive-sense single-stranded RNA genomes ~ 30 kilobases and over 10 open reading frames (ORFs)^3–5,7^. Two polypeptides, polyprotein 1a (pp1a) and pp1ab, are synthesized through ribosomal frameshift between ORF1a and ORF1b during translation^9,10^. In addition to the papain-like protease, the 3-chymotrypsin-like protease (3CLpro), also known as the main protease, is important for the posttranslational processing of SARS-CoV-2 polypeptides and the production of 16 non-structural proteins (nsps)^5,11,12^. The nsps play fundamental roles in replication, transcription, and virus recombination during an infection, where inhibiting the proteases will block the release of the nsps and inhibit the maturation and infectivity of SARS-CoV-2^3,5^. As a result, 3CLpro of SARS-CoV-2 is an attractive target for the design of broad-spectrum of antivirals against COVID-19^3,7,13,14^.

Among the Coronaviridae family, the 3CLpro substrate’s binding pocket is highly conserved with glutamine and leucine/methionine required at P1 and P2-positions, respectively, which correspond to the first and second residues before the cleavage site on the polypeptide substrate^10,13,15–18^. The 3CLpro cleaves the SARS-CoV-2 polyproteins at 11 sites “Leu Gln↓Ser Ala Gly,” which ↓ marks the cleavage site^10,13,15–18^. Multiple crystal structures of 3CLpro have been deposited in the Protein Data Bank, including the recently determined structure in complex with α-ketoamide inhibitors^13,18^.

**Figure 1:**
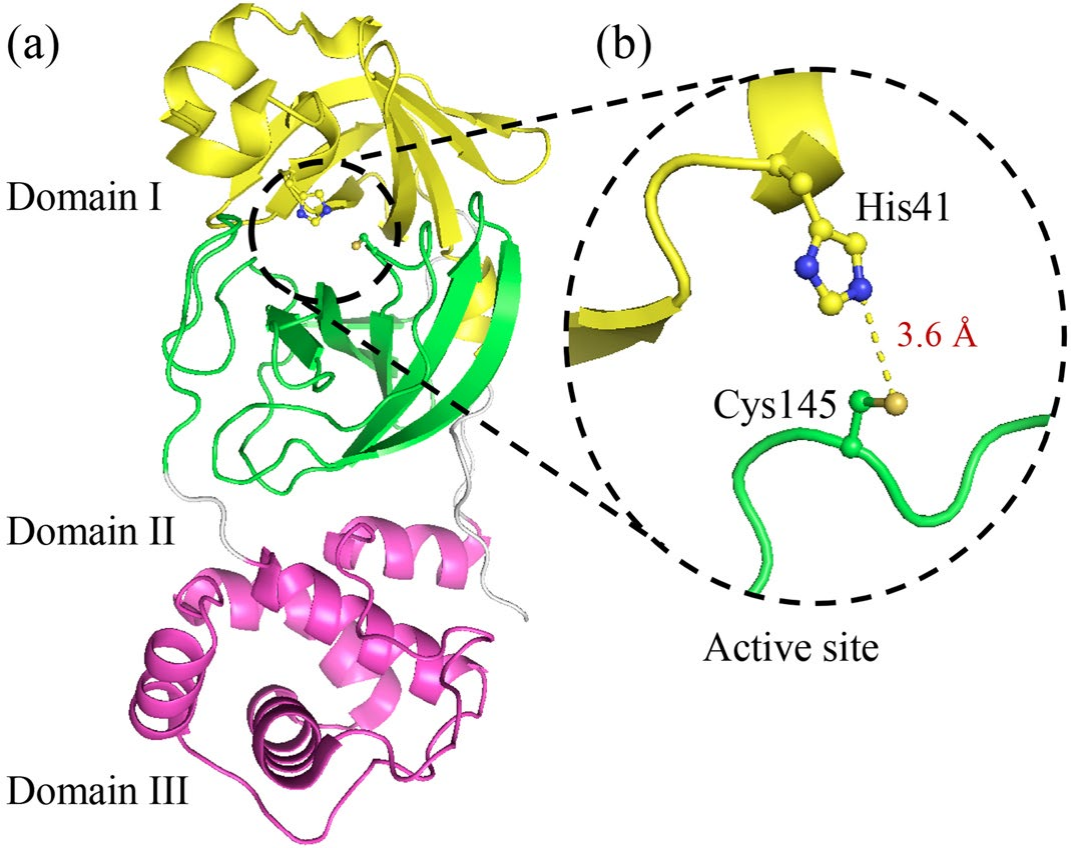
The crystal structure of 3CLpro. (**a**) Cartoon representation of the structural domains of 3CLpro of SARS-CoV-2 (PDB code 6Y2E). Domain I (residues 10–96) is shown in yellow, domain II (residues 102–180) in green, and domain III (residues 200–303) in pink. The active site is located at the interface between domains I and II. (**b**) The active site of 3CLpro showing the catalytic residue Cys145, which is part of domain II, is 3.6 Å from His41 of the catalytic dyad, which is part of domain I. The figure was prepared using PyMol (Schrodinger LLC).

The 3CLpro from Betacoronaviruses have identical structural folds, where the active site is highly conserved^13,15,16,18^. The monomer is split into three domains, with domains I (residues 10–96) and II (residues 102–180) having a five-stranded antiparallel β-barrel structure with a chymotrypsin-like folding scaffold (Fig. 1a)^13,18^. On the other hand, the C-terminal domain III (residues 200–303) has a five α-helices cluster that is connected to Domain II by a long loop (residues 181–199). Domain III of 3CLpro from SARS-CoV was identified to be important in the dimerization and formation of an active 3CLpro protease^19^. The active site of 3CLpro is at the interface between domains I and II, and different from the Ser–His–Asp triad of chymotrypsin, 3CLpro of SARS-CoV-2 has a catalytic Cys–His dyad (Fig. 1b)^13,18,20^. His41 and Cys145 are part of domains I and II, respectively, and they are 3.6 Å apart, which is an optimum distance to initiate hydrogen bonding interactions (Fig. 1b).

The first step in the catalytic reaction of 3CLpro is the deprotonation of the thiol side chain of Cys145 by His41 for its nucleophilic attack on the carbonyl carbon of glutamine of the polyprotein backbone. Upon its deprotonation, the thiolate ion of Cys145 attacks the peptide carbonyl carbon and forms a thiohemiketal that collapses into the thioester after the cleavage of the peptide bond and release of the C-terminal part of the polypeptide substrate^13,19,21^. Finally, a water molecule facilitates the hydrolysis of the thioester linkage, displacing Cys145 and releasing the N-terminal segment of the polypeptide substrate. The thioester linkage formation is an essential step in the catalytic mechanism of 3CLpro, and it is targeted in the development of antivirals^18^.

The biochemical and biophysical characterizations of 3CLpro are essential for the identification of optimum conditions to be used in the enzymatic assay for the screening of inhibitors that would be further developed as antivirals. Here, we characterize the thermodynamic and kinetic stability of 3CLpro from SARS-CoV-2 under different pH conditions and ionic strengths. 3CLpro was expressed in *E. coli* and purified to high purity. The secondary structural properties and native fold of the enzyme were confirmed by Circular Dichroism (CD) spectroscopy. 3CLpro was thermodynamically stable at a wide pH range of 6.0–10.0, with the highest stability recorded at pH 7.0. Interestingly, the presence of salts in the buffer solution decreased the thermodynamic stability of 3Lpro with magnesium chloride decreasing the stability further than sodium chloride. On the other hand, increasing the ionic strength of the buffer solution by increasing the concentration of NaCl or MgCl_2_ did not compromise the 3CLpro stability. The thermal kinetic stability of 3CLpro was also investigated, and the rate of thermal protein unfolding for 3CLpro was relatively slow at all pH values tested here with the lowest unfolding rate recorded at pH 10.0. However, the highest enthalpy of activation was recorded at pH 7.5. The data acquired here suggest biochemical conditions, including neutral or basic pH conditions in the absence of salt, to be used in the enzymatic assay of 3CLpro. The condition observed here will promote optimum conditions to set up high-throughput screening protocols for the identification of 3CLpro inhibitors to be developed as antiviral therapeutics against COVID-19.

## Results

### Purification and circular dichroism (CD) spectrum of 3CLpro

**Figure 2:**
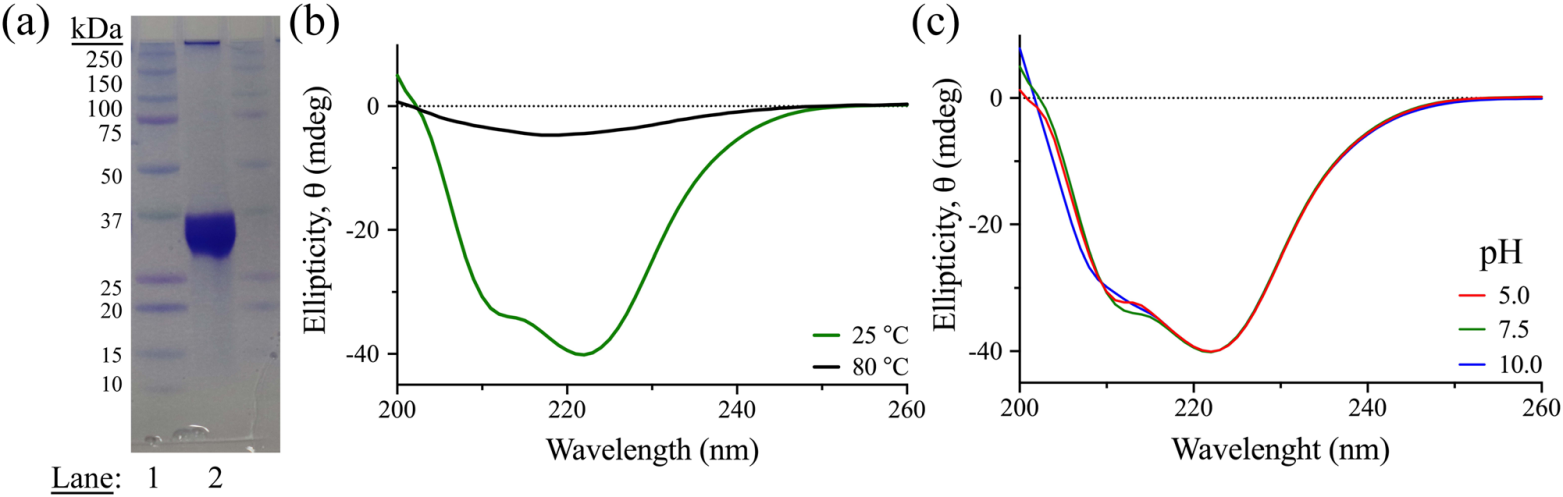
The SDS-PAGE analysis and far-UV CD scans of 3CLpro. (**a**) SDS-PAGE analysis of the 3CLpro with Coomassie-stain for visualization of the protein bands. The molecular weight marker and 3CLpro sample are loaded to lanes 1 and 2, respectively. (**b**) Far UV-CD spectra (280–200 nm) of 3CLpro at pH 7.5 for native (green) and denature (black) states collected at 25 °C and after 30 min incubation at 80 °C, respectively. Each spectrum is an average of five CD scans. (**c**) Far UV-CD spectra of native-state of 3CLpro collected at 25 °C in different pH values of 5.0 (red), 7.5 (green), and 10.0 (blue).

The 3CLpro gene was cloned into pET28b(+) vector, expressed in *E. coli,* and purified using Ni–NTA affinity and size exclusion chromatography to > 90% purity based on Coomassie staining SDS-PAGE analysis, where gel band densitometry was calculated using ImageJ software (Fig. 2a)^22^. The overall expression yield was high, with 5 mg of 3CLpro from one liter of terrific broth culture. The structural integrity of the 3CLpro was verified using far-UV circular dichroism (CD) analysis, with the spectrum exhibiting two ellipticity minima at 208 and 222 nm, which is similar to chymotrypsin-like fold with mixed α-helical and β-sheet structures (Fig. 2b)^23^. After thermal denaturation, the spectrum of 3CLpro changed significantly and diminished to a single broad peak with a minimum at ~ 215 nm. The far-UV CD spectrum of 3CLpro was also collected at different pH values to verify if the protein can tolerate a wide pH range. The spectrum of the native 3CLpro did not change at pH 5.0, 7.5, and 10.0, with limited perturbation of its secondary structure (Fig. 2c). The high secondary structural identity of 3CLpro at a different pH ensures proper characterization of the optimum biochemical and biophysical properties for the enzymatic reaction of 3CLpro, with limited interference on the overall protein structural integrity.

### The effect of pH on the thermodynamic stability of 3CLpro

**Figure 3:**
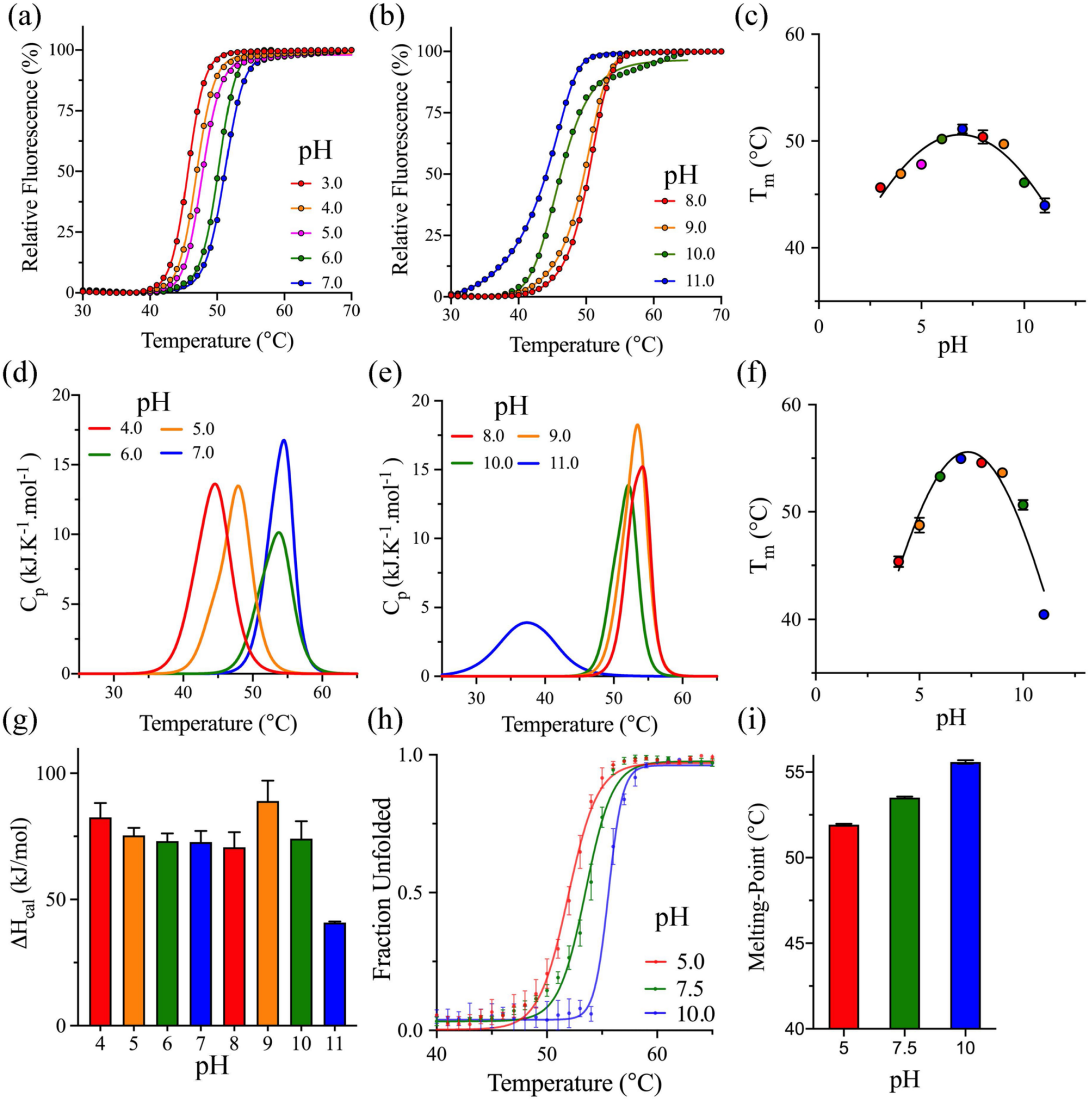
pH effect on the thermodynamic stability of 3CLpro. (**a–b**) DSF thermal scans of 3CLpro at different pH values from 3.0 to 11.0 in 100 mM phosphate buffer without the addition of salt. The DSF scans were collected in the presence of SYPRO Orange dye with an increase in florescence upon the binding of the hydrophobic surfaces of the protein to the dye. (**c**) pH dependence of *T*_m_ calculated from the midpoint of the thermal transitions of the DSF thermal scans of 3CLpro in panels A and B. Data are mean ± SD, n = 4. (**d–e**) DSC thermograms of 3CLpro in 100 mM phosphate buffer at different pH values from 4.0 to 11.0 in the absence of salt. Two separate DSC scans have been collected for each pH value with high reproducibility. (**f**) pH dependence of *T*_m_ calculated from the apex of the thermograms of 3CLpro from panels (**d**) and (**e**). (**g**) Bar plot of *ΔH*_cal_ at different pH values determined from the area under the thermographic peaks in panels (**d**) and (**e**). Data are mean ± SD, n = 2. (**h**) Thermal denaturation profiles of 3CLpro monitored by recording the ellipticity at 222 nm at pH 5.0 (red), 7.5 (green), and 10.0 (blue). The sample was heated at a rate of 1.0 °C/min, and T_m_ was calculated from the midpoint of the thermal transitions. (**i**) Bar plot of *T*_m_ calculated from the midpoint of the thermal transitions monitored by CD. Data are mean ± SD, n = 3.

Differential scanning fluorimetry (DSF) was used to determine the melting temperature (*T*_m_) from the global thermal unfolding of 3CLpro in the presence of a reporter dye, SYPRO Orange. The thermal unfolding transitions of 3CLpro were acquired at different pH values by monitoring the increase in fluorescence as the SYPRO Orange dye binds to the exposed protein’s hydrophobic core (Fig. 3a,b). The *T*_m_ was calculated at the midpoint of the DSF thermal transitions, with the highest *T*_m_ of 51.1 ± 0.4 °C recorded at pH 7.0 (Fig. 3c). Surprisingly, 3CLpro tolerated a wide range of pH values with relatively high thermodynamic stabilities, with an average value of ~ 50.4 ± 0.6 °C recorded between pH 6.0 and 9.0. The *T*_m_ decreased below pH 5.0 and above pH 10.0, with the lowest values of 45.6 ± 0.1 °C and 44.0 ± 0.7 °C recorded at pH 3.0 and 11.0, respectively. The ability of 3CLpro to tolerate a wide pH range of values was also confirmed by differential scanning calorimetry (DSC). The thermograms of 3CLpro acquired by DSC at different pH values exhibited a single transition with the *T*_m_ calculated at the apex of the melting peak and the calorimetric enthalpy (*ΔH*_cal_) determined from the area under the thermographic peak (Fig. 3d,e). Similar to DSF thermal scans, the 3CLpro was stable at a relatively wide pH range of 6.0–11.0, with the highest *T*_m_ of 55.0 ± 0.1 °C recorded at pH 7.0 (Fig. 3f). The amplitude of the DSC thermographic transitions did not change significantly at the different pH values tested here except at pH 11.0. As a result, the *ΔH*_cal_ at different pH values were relatively similar in value, with an average of 77 kJ/mol compared to 41 ± 0.4 kJ/mol at pH 11.0 (Fig. 3g). The *T*_m_ values determined from DSC were 4 °C higher than those calculated from DSF. The overall stability difference between the different techniques is expected since each relies on a different measurement strategy, where a reporter dye is included in DSF to monitor the global unfolding and exposure of the protein’s hydrophobic core. On the other hand, DSC directly measures the thermodynamic parameters acquired from unfolding the protein sample.

In addition to DSF and DSC analyses, the thermal unfolding transition of 3CLpro at different pH values was acquired using CD spectroscopy. The thermal denaturation curve of 3CLpro was monitored by CD spectroscopy with a large change in the CD signal at 222 nm, which was observed upon the denaturation of 3CLpro (Fig. 3h). The *T*_m_ was determined at the midpoint of the thermal unfolding transitions of 3CLpro after fitting the data to Boltzmann sigmoidal function. The *T*_m_ values of 3CLpro at pH of 5.0, 7.5, and 10.0 were 52.0 °C ± 0.1, 53.5 °C ± 0.1, and 56.0 °C ± 0.1, respectively (Fig. 3i). The *T*_m_ values acquired from CD thermal scans were in the same range as those acquired from DSC; however, the highest *T*_m_ value acquired from CD spectroscopy was at pH 10.0 compared with pH 7.0 from DSC analysis. The *T*_m_ calculated from CD thermal scans can be different from values acquired from other thermal analysis techniques. The signal in far-UV CD spectroscopy (190–240 nm) is primarily due to the absorption of the amide groups of the polypeptide backbone, where different secondary structures with specific dihedral angles contribute to the CD absorption. Therefore, the CD absorption signal is related to the protein’s secondary structure elements, which will make it different from other techniques, including DSC, with a direct measure of enthalpy values acquired upon protein unfolding. Overall, 3CLpro exhibited relatively high thermodynamic stabilities over a wide pH range as determined by different thermodynamic techniques tested here.

### The effect of metal ions and ionic strength on the thermodynamic stability of 3CLpro

**Figure 4:**
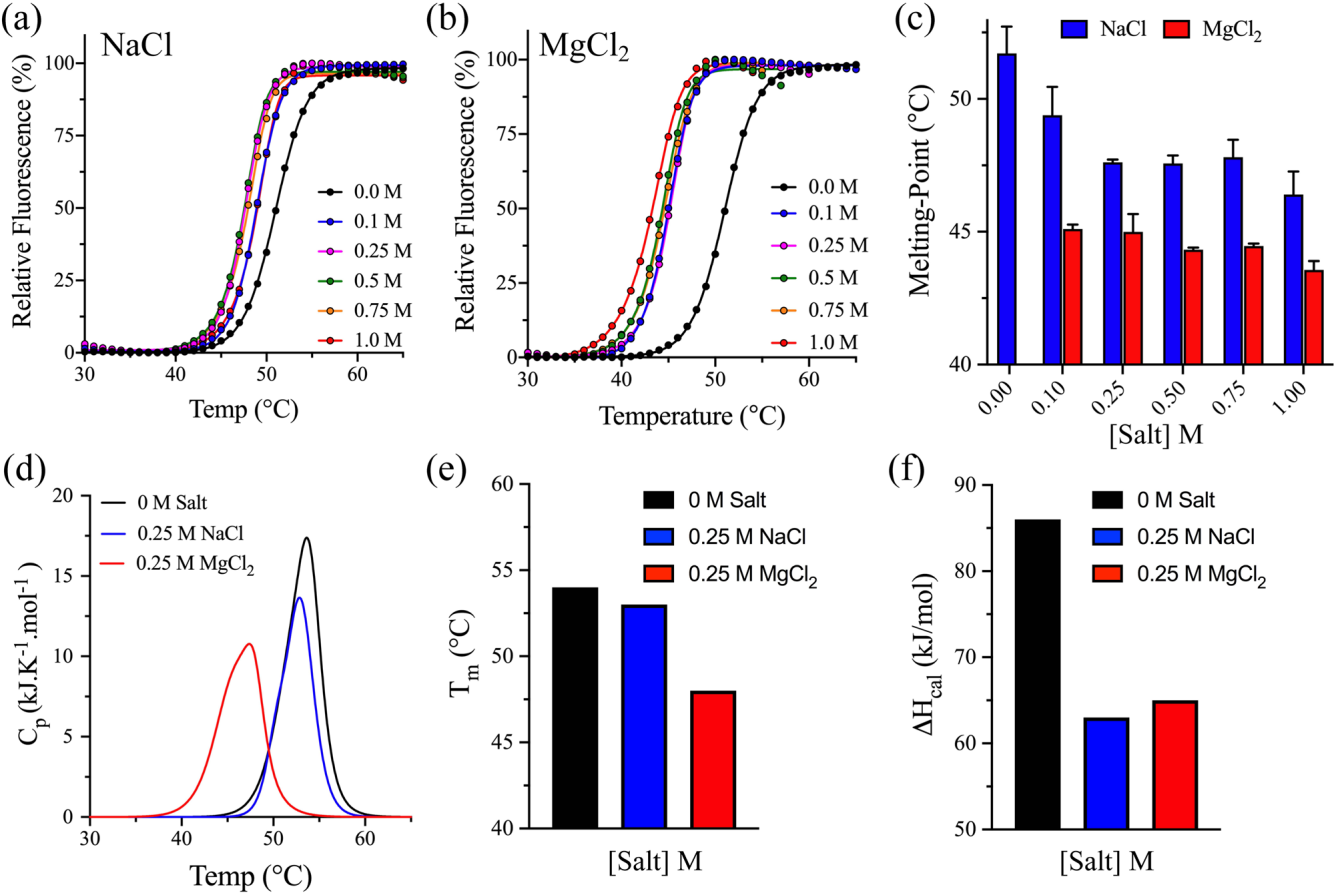
The effect of ionic strength on the thermodynamic stability of 3CLpro. (**a**,**b**) DSF thermal scans of 3CLpro in 50 mM phosphate buffer pH 7.5 at different concentrations of sodium chloride and magnesium chloride. The experimental conditions were similar to Fig. 3a,b. Data are mean ± SD, n = 4. (**c**) Bar plot of the ionic strength dependence on *T*_m_ of 3CLpro calculated from the DSF thermal transitions in panels (**a**) and (**b**). (**d**) DSC thermograms of 3CLpro in 50 mM phosphate buffer at pH 7.5 in the absence or presence of 0.25 M NaCl or 0.25 M MgCl_2_. (**e–f**) Bar plot of *T*_m_ and *ΔH*_cal_ of 3CLpro calculated from thermograms in panel (**d**) in the absence or presence of 0.25 M NaCl or 0.25 M MgCl_2_.

Similar to the pH effect, the influence of salts and ionic strength, including monovalent (Na^+^) and divalent (Mg^2+^) cations, was investigated for the thermodynamic stability of 3CLpro. DSF was used to acquire the thermal unfolding transitions of 3CLpro at pH 7.5 in the absence or presence of sodium or magnesium chloride by monitoring the increase in the SYPRO Orange fluorescence signal (Fig. 4a,b). The *T*_m_ at the midpoint of the transition decreased from 51.3 ± 0.8 °C in the absence of salt to 48.8 ± 0.1 °C and 45.1 ± 0.2 °C in the presence of 0.1 M NaCl and 0.1 M MgCl_2_, respectively (Fig. 4c). The thermal unfolding transition of 3CLpro was acquired at different concentrations of NaCl and MgCl_2_ to investigate the effect of the ionic strength on the stability of 3CLpro. The change in *T*_m_ was minimum upon increasing the salt concentration with average values of 47.7 ± 0.7 °C and 44.6 ± 0.6 °C at different concentrations from 0.25 M to 1.0 M for NaCl and MgCl_2_, respectively (Fig. 4c).

The stability of 3CLpro was also investigated using DSC in the presence of 0.25 M NaCl and 0.25 M MgCl_2_ at pH 7.5. Similar to DSF analysis, the DSC thermographic peak shifted to low temperature in the presence of slat with a larger destabilization effect for magnesium compared to sodium (Fig. 4d). The *T*_m_ of 3CLpro decreased from 54 °C in the absence of salt to 53 °C and 48 °C in the presence of 0.25 M NaCl and 0.25 M MgCl_2_, respectively (Fig. 4e). However, the *ΔH*_cal_ slightly decreased from 86 kJ/mol in the absence of salt to 63 kJ/mol and 65 kJ/mol in the presence of NaCl and MgCl_2_, respectively (Fig. 4f). Overall, the thermodynamic stability of 3CLpro was not affected by increasing the ionic strength upon increasing the salt concentration. However, divalent metal cations (Mg^2+^) destabilized the thermodynamic stability of 3CLpro more than monovalent cations (Na^+^).

### Denaturation kinetics of 3CLpro

**Figure 5:**
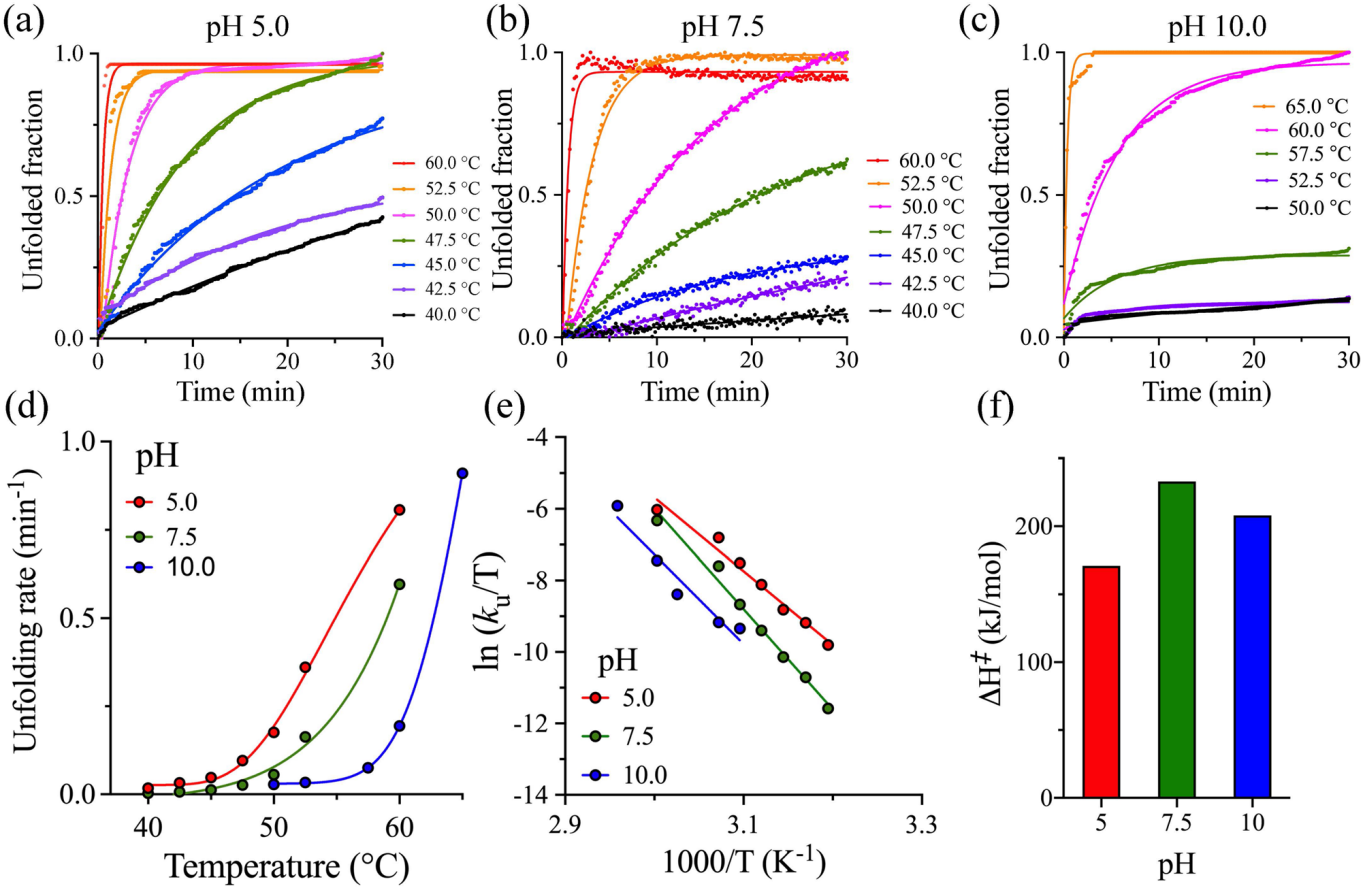
The thermal kinetic stability of 3CLpro. (**a–c**) Time courses of thermal denaturation of 3CLpro at different temperatures from 40.0 to 65.0 °C at different pH values of 5.0, 7.5, and 10.0. The change in ellipticity was continuously monitored at 222 nm for 30 min. Points on the graphs represent experimental data, and lines are the theoretical fit of the data to a single exponential function. The rate of unfolding (*k*_U_) was calculated from the linear portion of the graphs. (**d**) *k*_U_ plotted as a function of temperature at different pH values. The 3CLpro shows the highest unfolding kinetics at pH 5.0. On the other hand, pH 10.0 displayed the highest kinetic stability with a slow rate of unfolding with incubation temperature lower than 57.5 °C. (**e**) Eyring plot of the temperature dependence of *k*_U_ for 3CLpro at different pH values. Points on the graph are experimental data, and lines are the linear least-square regressions to experimental data. Changes in the slope of the lines are observed as a result of variation in Δ*H*^*‡*^. (**f**) Bar plot of Δ*H*^*‡*^ of the temperature dependence of the unfolding rate constant at different pH values with the highest Δ*H*^*‡*^ observed at pH 7.5.

Isothermal denaturation was used to determine the thermal kinetic of unfolding for 3CLpro by monitoring the protein unfolding rate at pH values of 5.0, 7.5, and 10.0. The unfolding rate constant (*k*_U_) was calculated at different incubation temperatures 40–60 °C from the linear slope of the enzyme denaturation signal monitored at 222 nm in a CD spectrophotometer (Fig. 5a–c). The unfolded fractions of 3CLpro were determined from a comparison of the fully unfolded and native folded-states. 3CLpro displayed the slowest *k*_U_ value at pH 10.0 with incubation temperatures up to 50 °C, which increased upon increasing the incubation temperatures (Fig. 5d). The lowest kinetic stability for 3CLpro was recorded at pH 5.0, with protein unfolding recorded at an incubation temperature of 40 °C (Fig. 5a). The enthalpy of activation (Δ*H*^*‡*^) was determined from the slope of lines in the Eyring plot (ln(*k*_U_/T) versus 1/T) of the temperature dependence of the unfolding rate constant (Fig. 5e). The kinetic of unfolding data at different pH values were obtained over a broad temperature range of 40–60 °C, where the Eyring plots show an increase in the *k*_U_ as a function of temperature. The linearity in Eyring plots indicates no significant heat capacity change between the folded ground state and the transition state of the thermal unfolding of 3CLpro. The Eyring equation, shown below, was used to interpret the temperature dependence of the second-order rate constants of 3CLpro unfolding.

$$ln\frac{{k_{U} }}{T} = \frac{{ - \Delta H^{\ddag } }}{R}\frac{1}{T} + ln\frac{{k_{B} }}{h} + \frac{{\Delta S^{\ddag } }}{R},$$

where *k*_B_ is Boltzmann’s constant, *h* is Planck’s constant, *R* is the gas constant, *T* is the absolute temperature, and Δ*S*^*‡*^ is the entropy of activation. A noticeable change is observed in the slopes of the Eyring plots, which indicate variations in Δ*H*^*‡*^ with pH, especially at pH 7.5. The Δ*H*^*‡*^ was determined from the slope of lines ($\frac{{ - \Delta H^{\ddag } }}{R}$), which was 171 kJ/mol, and 233 kJ/mol, and 208 kJ/mol at pH 5.0, 7.5, and 10.0, respectively (Fig. 5f). As a result, 3CLpro displayed the highest kenotic stability at pH 7.5 even though the rate of protein unfolding was slower at pH 10. The Δ*H*^*‡*^ represents the energy barrier between the folded ground state and the partially unfolded transition state^24^.

## Discussion

In the fight against COVID-19 and the spread of SARS-CoV-2, the discovery of antiviral drugs and the development of therapeutics are of great importance. One of the key enzymes in the processing of new virus particles of SARS-CoV-2 is the main protease, 3CLpro, which regulates replicase polyprotein processing and the release of functional proteins during virus maturation. As a result, 3CLpro makes an attractive target in the development of antiviral therapeutics against COVID-19. In this study, we demonstrated the expression and biochemical and biophysical properties of 3CLpro from SARS-CoV-2 to facilitate optimum conditions for drug screening and development.

Different thermal analytical techniques have been implemented here to determine the fold stability and thermodynamic properties of 3CLpro at different pH values and in the presence or absence of monovalent (Na^+^) or divalent (Mg^2+^) metal cations. The melting temperature of 3CLpro was relatively consistent at different pH values of 6.0–9.0, with an average of ~ 50 °C from DSF analysis and ~ 54 °C recorded by DSC and CD spectroscopy. The variation in *T*_m_ values from different thermal analytical techniques used here is expected due to the different measurement strategies applied especially for DSF analysis that utilizes a reporter dye to monitor the global unfolding of 3CLpro compared with DSC that directly measures the thermodynamic properties of unfolding or CD spectroscopy that monitors changes in the secondary structure of the protein. Previously, DSC analysis on 3CLpro from the 2003 SARS-CoV revealed *T*_m_ values of 52.5 °C and 56.50 ± 0.03 °C at pH 7.4^25–27^, which was in the range of the value of 55.0 ± 0.1 °C at pH 7.0 acquired here. However, the enthalpy value of 3CLpro from SARS-CoV has not been reported. The similar *T*_m_ values of 3CLpro from SARS-CoV and SARS-CoV-2 are due to the high structural similarity of the protease among the different coronaviruses.

Interestingly, the DSC thermograms at the different pH values except for pH 11.0 exhibited a similar amplitude that yields similar *ΔH*_cal_ values. The similarity in the calorimetric enthalpy of 3CLpro at different pH values is an indication of similar bonding interactions that maintain the structural fold of the protein. It has been shown previously that proteins with dominated hydrophilic structural interactions exhibit an exothermic positive thermographic peak compared with hydrophobic interactions that exhibit an endothermic negative thermographic peak^28^. The reduced *ΔH*_cal_ value at pH 11.0 is an indication of a structural fold with dominated hydrophobic interactions compared to the hydrophilic character of the protein at the other pH values tested here.

From DSF analysis, the addition of salts decreased the thermal stability of 3CLpro from COVID-19, with the *T*_m_ decreased by 3.6 °C and 6.7 °C in the presence of NaCl and MgCl_2_, respectively. A similar result was observed from DSC analysis, with a decrease in the *T*_m_ of 3CLpro by 6.0 °C in the presence of MgCl_2_. However, NaCl decreased the *T*_m_ by 1.0 °C. The *ΔH*_cal_ also decreased by ~ 22 kJ/mol in the presence of NaCl or MgCl_2_. The destabilization of the thermal stability of 3CLpro was dependent on the type of metal cations, where divalent (Mg^2+^) cations had a more pronounced destabilization effect on the thermodynamic stability of 3CLpro compared with monovalent (Na^+^) cations. On the other hand, the thermal stability of 3CLpro was independent of the ionic strength of the buffer solution, where increasing the concentration of sodium or magnesium chloride did not further reduce the thermal stability of 3CLpro. The reduced thermal stability of 3CLpro in the presence of salt may be associated with the destabilization of salt bridges, where it has been shown that ion-pair networks in proteins are responsible for their increased thermal stabilities^29,30^. The monovalent cations on sodium can neutralize negatively charged residues and interrupt the formation of salt bridges. Still, it cannot form new cross-linked interactions where the higher charge density of divalent cations leads to a higher accumulation and interaction with negatively charged and polar amino acid residues. Therefore, in addition to its ability to disrupt ionic interactions that stabilize the protein structure, the cross-linking effect of divalent cations allows for the formation of new salt bridges, which may enhance protein aggregation and further contribute to the destabilization effect of magnesium compared with sodium on the thermodynamic stability of 3CLpro^30^.

The thermal kinetic stability of 3CLpro was recorded at different pH values, where the rate of protein unfolding was monitored by CD spectroscopy at different incubation temperatures. The lowest rate of unfolding for 3CLpro was recorded at pH 10.0. The enthalpy of activation (Δ*H*^*‡*^) calculated from the slope of Eyring plots was positive at all pH values tested here due to the disruption of noncovalent bonding interactions on the protein during the transition from the folded (ground) state to the activated (transition) state. However, the highest Δ*H*^*‡*^ was recorded at pH 7.5 with a value close to that at pH 10.0, where the later recorded the slowest unfolding rate. The kinetic stability is related to the activation energy, and it is proportional to the size of the kinetic barrier separating the native and unfolded states, where an increase in kinetic stability is proportional to the increase in the energy barrier between the folded ground state and denatured or partially unfolded transition state^24^. Overall, the highest kinetic stability of 3CLpro was recorded at a basic pH value with relatively similar Δ*H*^*‡*^ at pH 7.5 and 10.0.

The biochemical and biophysical properties of 3CLpro explored here highlight high thermodynamic and kinetic stabilities at wide pH values with preference to more basic pH values between pH 7.5 and 10.0. However, the presence of salts and especially divalent metal cations destabilized the thermodynamic stability of 3CLpro with no effect observed upon increasing the ionic strength. Due to the high structural similarity of 3CL proteases of SARS-CoV and SARS-CoV-2 may be reasoned for the screening and identification of inhibitors of 3CLpro to be used in the development of new antiviral therapeutics to limit the spread of SARS-CoV-2^3,7,13,15,16,18^. The biochemical and biophysical properties explored here would facilitate the setup of optimum conditions for the 3CLpro enzymatic assay.

## Material and methods

### Expression and purification of 3CLpro

The recombinant 3CLpro gene was introduced) into pET28b(+) bacterial expression vectors by GenScript Inc (Piscataway, NJ. The expression of the Hisx6-tagged human 3CLpro protein was performed in *E. coli* BL21-CodonPlus-RIL (Stratagene). The inoculated culture (2–6 L) was grown in Terrific Broth (TB) at 30 °C until the *A*_600_ reached 0.8 in the presence of 100 mg/L kanamycin and 50 mg/L chloramphenicol. The temperature was then lowered to 15 °C and the expression was induced overnight with 0.5 mM IPTG. The cells were harvested by centrifugation at 12,000×*g* at 4 °C for 10 min in an Avanti J26-XPI centrifuge (Beckman Coulter Inc.), then resuspended in lysis buffer (20 mM Tris pH 7.8, 150 mM NaCl, 5 mM imidazole, 3 mM βME, and 0.1% protease inhibitor cocktail from Sigma-Aldrich: P8849). Cell lysis was carried out using sonication on ice, then centrifuged at 40,000×*g* for 45 min at 4 °C. The supernatant was loaded on a ProBond Nickel-Chelating Resin (Life Technologies) previously equilibrated with binding buffer (20 mM Tris pH 7.5, 150 mM NaCl, 5 mM imidazole, and 3 mM βME) at 4 °C. The resin was washed with 10 column volumes (cv) of binding buffer, followed by 15 cv of washing buffer (20 mM Tris pH 7.5, 150 mM NaCl, 25 mM imidazole, and 3 mM βME). The His-tagged 3CLpro enzyme was eluted from the column with 20 mM Tris, pH 7.5, 150 mM NaCl, 300 mM imidazole, and 3 mM βME in 1 mL aliquots. Finally, the Ni-column fractions containing 3CLpro were loaded onto a HiLoad Superdex 200 size-exclusion column (GE Healthcare) using an AKTA purifier core system (GE Healthcare). The column was pre-equilibrated with filtration buffer (20 mM Hepes pH 7.5, 150 mM NaCl, and 0.5 mM TCEP). The final protein was collected and concentrated to ~ 150 μM based on Bradford assay, and the sample purity was assessed via SDS–PAGE (Fig. 2a).

### Differential scanning calorimetry (DSC) and differential scanning fluorimetry (DSF)

The thermodynamic stability of the 3CLpro was measured using Nano-DSC (TA Instruments) that has been calibrated using chicken egg white lysozyme, a known external standard for Nano DSC as part of the test kit (602,198.901) from TA instrument. The thermogram was acquired at 30 μM protease in different pH values utilizing 100 mM phosphate buffer. The sample was heated at a scan rate of 1 °C/min from 15 to 75 °C at 3 atm pressure. The background scans were obtained by loading degassed buffer in both the reference and sample cells and heated at the same rate. The DSC thermograms were corrected by subtracting the corresponding buffer baseline and converted to plots of excess heat capacity (*C*_p_) as a function of temperature. The melting point (*T*_m_) was determined at the maximum temperature of the thermal transition, and the calorimetric enthalpy (*ΔH*_cal_) of the transitions was estimated from the area under the thermal transition using Nano Analyzer software from TA instruments. Additional DSC scans were collected at different ionic strength in the presence or absence of 250 mM NaCl or 250 mM MgCl_2_ in 50 mM phosphate buffer at pH 7.5.

In addition to DSC analysis, the T_m_ of 3CLpro was determined using DSF measurements in the presence of SYPRO Orange fluorescent reporter dye using a real-time QPCR instrument (Mx3005P QPCR system, Agilent Technologies, La Jolla, CA). The measurements were conducted in a 96-well thin-walled PCR microplate (BioRad, Cat. No. 223 94444) with excitation and emission at 492 nm and 610 nm, respectively. The thermal scans were acquired at a concentration of 7.5 μM for 3CLpro in the presence of 3X SYPRO Orange dye at different pH values utilizing 50 mM phosphate buffer. The fluorescence measurements of the protein unfolding signals were collected from 25 to 80 °C at a fixed temperature ramp rate of 1 °C/min. The QPCR instrument is equipped with a Peltier-based thermal system for uniform ramping and thermal accuracy to ensure reproducibility of the data that were fitted to a Boltzmann sigmoidal function and the *T*_m_ was calculated at the middle of the transition using the Excel add-on package XLfit (IDBS limited, Bridgewater, NJ, U.S.A.) as described previously^28^. Similar to DSC, the *T*_m_ of 3CLpro was measured at different ionic strength in the presence or absence of 250 mM NaCl or 250 mM MgCl_2_ in 50 mM phosphate buffer at pH 7.5.

### Circular dichroism (CD) spectra and kinetic stability analysis

The CD spectra of 3CLpro were collected in a 100 mM phosphate buffer at pH 5.0, 7.5, and 10.0 from 190–260 nm at 10 nm/s scanning speed on a Chirascan CD spectrometer (Applied Photophysics), calibrated with aqueous camphor-10-sulfonic acid (CSA). The protease concentration utilized for CD analysis was 30 μM and measured using a 1 mm quartz cuvette and 1 nm bandwidth at 25 °C. On the other hand, the thermal denaturation profiles of 3CLpro were determined by the heat induced conformational transition of native to the denatured state by monitoring the ellipticity changes at 222 nm while the sample temperature was increased at a rate of 1.0 °C/min. The same sample condition and instrumentation set up were utilized as in the CD spectrum analysis. The thermal transition measurements were conducted at different pH values and normalized to fraction unfolded (*F*_Unf_) using the following equation.

$$F_{unf} = \frac{{\theta - \theta_{N} }}{{\theta_{D} - \theta_{N} }},$$

where *θ* is ellipticity of protein at a specific time, and *θ*_N_ and *θ*_D_ are the ellipticities of native and denatured states, respectively. *θ*_N_ of the native state was obtained before temperature incubation of 3CLpro, and *θ*_D_ was obtained at the end of the measurement and after incubating the protein at 80 °C for 1 h. The data were fitted to a Boltzmann sigmoidal function and the T_m_ was calculated at the middle of the transition using the Excel add-on package XLfit (IDBS limited, Bridgewater, NJ, U.S.A.) as described previously^28^.

Finally, the thermal kinetic stability of 3CLpro was determined using isothermal denaturation analysis to calculate the rate of thermal unfolding after incubating the protein sample in 100 mM phosphate buffer at different temperatures 40–65 °C and pH values of 5.0, 7.5, and 10.0. The ellipticity (*θ*) at 222 nm was continuously collected for 30 min and utilized to calculate the *F*_Unf_ as described above. The rate of protein unfolding (*k*_U_) was determined from the slope of the line after fitting the data to a straight line.
